# Effects of Temperature and Exposure Duration on Energy Substances and Antioxidant Enzymes in *Riptortus pedestris* (Hemiptera: Alydidae)

**DOI:** 10.3390/insects17050506

**Published:** 2026-05-15

**Authors:** Ke Song, Liyan Zhang, Xiaofeng Li, Sizhu Zhao, Wendi Qu, Meng-Lei Xu, Jing Yang, Yu Gao

**Affiliations:** 1 College of Plant Protection, Jilin Agricultural University, Changchun 130118, China; 2 College of Food Science and Engineering, Jilin University, Changchun 130062, China; 3 Key Laboratory of Soybean Disease and Pest Control, Ministry of Agriculture and Rural Affairs, Changchun 130118, China; 4 Department of Life Sciences, Xinzhou Normal University, Xinzhou 034000, China

**Keywords:** antioxidant enzyme, energy substance, heat stress, physiological response, thermoregulation

## Abstract

Soybean (*Glycine max*) is a vital food and oil crop in China, yet its yield and quality are severely threatened by piercing–sucking damage caused by *Riptortus pedestris* (Hemiptera: Alydidae) to soybean pods. Under global climate warming and expanded soybean cultivation, temperature has become a key environmental factor driving the spread of and aggravated damage caused by *R. pedestris*. We investigated the effects of temperature (32, 36, 40, 42, and 44 °C) and exposure duration (1–4 h) on the energy substances and antioxidant enzyme activities in adult *R. pedestris*. These two factors also had significant effects on the pest’s energy substances and antioxidant defense. Under short-term high-temperature stress, the water loss rate and fat, total sugar, and glycogen contents increased significantly, while protein content showed a fluctuating upward trend, with distinct sexual differences in these responses; the water loss and energy substance levels within the lethal high-temperature range, around 44 °C, were generally higher than those in the sublethal range (36–42 °C). *R. pedestris* showed physiological changes consistent with enhanced heat tolerance and adaptability, including water balance regulation, carbohydrate and lipid accumulation, and modulation of protein synthesis and degradation. In the sublethal high-temperature range, antioxidant enzyme activity patterns were altered, and SOD activity was increased; meanwhile, the MDA content also rose, and POD and CAT activities decreased. In the lethal high-temperature range, the overall antioxidant enzyme activities were lower than in the suitable temperature range, with the POD activities and MDA content still rising. These results suggest that the dynamic adjustment of antioxidant enzyme activities may contribute to alleviating oxidative damage and rapid adaptation to temperature-induced oxidative stress in *R. pedestris*. These findings indicate that *R. pedestris* possesses physiological plasticity to cope with sublethal heat stress through metabolic reallocation and antioxidant defense activation, but extreme temperatures cause severe physiological disruption. This study provides insights into the thermal biology and heat resistance mechanisms of this pest under climate warming scenarios.

## Simple Summary

Soybean production in China is under severe threat from the green stem and leaf retention disorder, which is mainly caused by piercing–sucking damage induced by *Riptortus pedestris* (Hemiptera: Alydidae). With climate warming and expanding soybean cultivation, temperature has become a core factor driving the spread and intensified destructive effects of this pest. Through short-term high-temperature stress experiments (32, 36, 40, 42, and 44 °C, 1–4 h), this study found that enhanced heat tolerance in *R. pedestris* was associated with the regulation of water balance, the accumulation of energy substances, and changes in protein content, with significant differences in physiological responses between females and males. Meanwhile, high temperatures were accompanied by the disruption of the antioxidant enzyme system and subsequent oxidative damage, but dynamic adjustments in enzyme activities suggested a compensatory response that could partially repair the damage. These results not only reveal the physiological mechanisms of *R. pedestris* in adapting to high temperatures but may also contribute to understanding its potential population spread under climate change and may inform future studies on developing targeted control strategies, which could help protect soybean crops more effectively.

## 1. Introduction

Global warming has intensified the frequency and severity of extreme heat events, posing unprecedented challenges to insect survival. As ectothermic organisms, insects rely entirely on behavioral and physiological adjustments to cope with thermal fluctuations, making them particularly vulnerable to temperature extremes [1,2]. Short-term high-temperature stress—defined as brief exposure to temperatures exceeding the optimal range—can trigger acute physiological dysfunction or mortality. This phenomenon provides a valuable model for dissecting rapid thermal responses in insects [3,4]. Such thermal perturbations compromise enzyme function, membrane integrity, and metabolic homeostasis, cascading into reduced fitness (slower development, impaired reproduction, and elevated mortality) and consequently constraining population viability and geographic distribution [5,6].

The bean bug *Riptortus pedestris* (Hemiptera: Alydidae), a major pest of leguminous crops, exemplifies these climate-driven impacts: heat waves disrupt its population dynamics and metabolic homeostasis [7]. Previous research on thermal biology of *R. pedestris* has predominantly examined constant and fluctuating temperature regimes, revealing their effects on development, reproduction, and respiration [8]. These studies demonstrate that, while moderate warming (within 19–31 °C) accelerates development and enhances fecundity, thermal extremes (≤16 °C or ≥36 °C) cause developmental arrest and prevent life cycle completion [9]. The optimal range for population growth is 24–28 °C, with peak fecundity observed at 24 °C [10,11]. Despite these advances, a critical gap remains: how do adult *R. pedestris* coordinate energy substances and antioxidant defenses during acute heat stress? This area of the field is poorly understood. This knowledge deficit limits our ability to predict population responses to increasingly frequent heat waves. Short-term high-temperature stress has been associated with three major types of responses in insects: (i) the disruption of water balance through evaporative cooling, with lethal outcomes when dehydration exceeds critical thresholds [12,13,14,15,16,17]; (ii) metabolic reprogramming, characterized by altered enzyme activities and substrate switching (e.g., from carbohydrates to lipids) to meet elevated energy demands [18,19,20,21,22]; and (iii) oxidative stress, necessitating the upregulation of antioxidant enzymes to scavenge reactive oxygen species and prevent lipid peroxidation (measured as malonic aldehyde accumulation) [23].

To investigate these responses, we exposed adult *R. pedestris* to ecologically relevant heat stress (32, 36, 40, 42, and 44 °C for 1–4 h) and measured the three key response variables: (i) the water loss rate, (ii) energy substrate levels, and (iii) antioxidant enzyme activities. Considering temperature and duration as factors, we seek to define the thermal tolerance limits and plasticity of this pest, providing insights critical for predicting population adaptability under future climate scenarios.

## 2. Materials and Methods

### 2.1. Test Insect Source

The various life stages of *R. pedestris* were collected from a soybean field (Xinzhou, Shanxi Province) and transferred to a laboratory breeding room to establish a stock population. The rearing conditions were maintained at 24 ± 1 °C, 85% ± 5% relative humidity, and a photoperiod of 16:8 h (L:D). The insects were fed on soybean plants and water-soaked soybeans until adulthood [10,24]. For the experiments, 3-day-old adults were used. Males and females were separated, and individuals were randomly assigned to different treatment groups.

The temperature × exposure duration was used for the heat stress survival assay. The control treatment was set at the rearing temperature of 24 ± 1 °C, which represents the optimal temperature for the species’ development and is commonly experienced under field conditions in soybean-growing regions. The experimental heat treatments were set at 32 ± 1 °C, 36 ± 1 °C, 40 ± 1 °C, 42 ± 1 °C, and 44 ± 1 °C, with exposure durations of 1, 2, 3, and 4 h. These temperatures cover a range from moderate to lethal heat waves that can occur during summer in temperate and subtropical agricultural ecosystems, thereby reflecting realistic thermal extremes. Subsequent measurements of specific physiological indicators were performed according to established protocols [20,25,26].

### 2.2. Test Instruments and Equipments

An intelligent artificial climate chamber (Model PGX-500D, Zhongyi Guoke (Beijing) Technology Co., Ltd., Beijing, China), electric heating blast dryer (Model 101-IBS, Shanghai Lichen Xi Instrument Technology Co., Ltd., Shanghai, China), multi-sample tissue grinder (Model WD-9419A, Beijing Liuyi Biotechnology Co., Ltd., Beijing, China), electronic balance (Model AL-204, Mettler Toledo Instruments (Shanghai) Co., Ltd., Shanghai, China), multifunctional centrifuge (Model Centrifuge 5430R, Eppendorf China Co., Ltd., Shanghai, China), fixed homogenizer (Model MX-F, Sciogex, Rocky Hill, CT, USA), microplate reader (Model HBS-1096C, Nanjing Detie Biotechnology Co., Ltd., Nanjing, China), and ground glass test tubes, and centrifuge tubes were used.

### 2.3. Reagents and Materials

Chloroform and Methanol (Analytical Reagent grade) were purchased from Tianjin Xinbote Chemical Co., Ltd., Tianjin, China. Phosphate-Buffered Saline (PBS, pH = 7.4) was purchased from Beijing Lanjieke Technology Co., Ltd.; 0.90% Physiological Saline was purchased from Jilin Province Xinbang Pharmaceutical Co., Ltd., Changchun, China; Glycogen, Total Sugar, and Total Protein Assay Kits (Product Nos.: A043-1-1, A156-1-1, and A045-2-2) were purchased from the Nanjing Jiancheng Bioengineering Institute, Nanjing, China. A superoxide dismutase (SOD) assay kit (Reagent 1, Product No. S0101S), catalase (CAT) assay kit (Reagent 2, Product No. S0051), total antioxidant capacity (T-AOC) assay kit (Reagent 3, Product No. S0116), and malonic aldehyde (MDA) assay kit (Reagent 4, Product No. S0131S) were purchased from Shanghai Beyotime Biotechnology Co., Ltd., Shanghai, China. A peroxidase (POD) assay kit (Reagent 5, Product No. JL45377) was purchased from Shanghai Jianglai Biotechnology Co., Ltd., Shanghai, China. A PBS solution (pH = 7.4) was purchased from Beijing Lanjieke Technology Co., Ltd., Beijing, China. All procedures were performed following the manufacturer’s instructions, validated for insect tissues.

### 2.4. Treatment Methods

Based on the results of the heat stress survival test on *R. pedestris*, the control treatment was set at 24 ± 1 °C. The experimental treatments were set at 32 ± 1 °C, 36 ± 1 °C, 40 ± 1 °C, 42 ± 1 °C, and 44 ± 1 °C, with exposure durations of 1, 2, 3, and 4 h. Subsequent experiments were conducted according to the specific indicators to be measured.

### 2.5. Determination Methods of Energy Substances

#### 2.5.1. Method of Water Loss Rate Measurement

Self-made perforated 1.5 mL centrifuge tubes were used to ensure moisture loss from the test insects. One 3-day-old adult was placed in each centrifuge tube, with 10 replicates. The tubes containing the insects were weighed before the experiment (*w*_1_). After treatment under different temperature and time conditions, they were weighed again (*w*_2_).

(1) $$W=(w_{1}-w_{2})/w_{1}\times 100\%$$

where *w*_1_ is the mass of the insect body containing water, *w*_2_ is the mass of the insect body after drying, and *W* is the water loss rate of the insect body [27].

#### 2.5.2. Method of Fat Content Determination

Ten treated insects from each treatment were weighed and then placed in a 60 °C electric blast drying oven for 48 h to obtain the dry weight (*M*_1_), which served as the sample dry weight. The insect bodies were ground to a homogenate using a chloroform–methanol extraction solution (Vchloroform:Vmethanol = 2:1), followed by centrifugation to collect the supernatant. The extraction solution was added to the precipitate again, followed by another centrifugation at 4000 rpm for 10 min to collect the supernatant. The remaining precipitate was dried in a 60 °C constant-temperature oven for 24 h and then weighed (*M*_2_). The experiment was repeated three times. The fat content was calculated using Formula (2) [28].

(2) $$M=M_{1}-M_{2}$$

where *M*_1_ is the mass of the insect body containing fat, *M*_2_ is the mass of the insect body without fat, and *M* is the fat content of the insect body.

#### 2.5.3. Method of Total Sugar Content Determination

Ten treated adult insects (five males and five females) from each treatment were ground into a fine powder. Reagent 1 refers to 5% phenol. Reagent 2 refers to concentrated sulfuric acid. Reagent 3 refers to the phenol–sulfuric acid chromogenic working solution. Exactly 0.1 g of the powder was accurately weighed using a balance and placed into a test tube; 1.5 mL of distilled water and 1 mL of Reagent 1 were added to the tube, the contents of which were mixed well and then heated in a boiling water bath for 30 min. After the tube had cooled to room temperature, 1 mL of Reagent 2 was added and mixed in well, and the volume was made up to 10 mL. The mixture was centrifuged at 8000 rpm for 10 min at 4 °C, and the supernatant was collected for analysis. The supernatant (μL) was mixed with Reagent 3 (μL) in a 1:1 ratio, heated in a boiling water bath for 10 min, and cooled. Then, 30 μL of the mixture was taken and prepared according to the reaction system. The absorbance (OD value) was measured at a wavelength of 540 nm (Table S1).

#### 2.5.4. Method of Glycogen Content Determination

Ten treated adult insects (five males and five females) were placed into a test tube. Then, 0.75 mL of phosphate-buffered saline (PBS, 0.1 mol/L, pH 7.4) was added to the tube, mixed thoroughly, and heated in a 95 °C water bath for 20 min. After the tube had cooled to room temperature, the contents were mixed again and diluted to a final volume of 5 mL with PBS. The mixture was centrifuged at 8000 rpm for 10 min at 4 °C. Subsequently, 200 μL of the supernatant was taken, and its absorbance (OD value) was measured at a wavelength of 620 nm (Table S1).

#### 2.5.5. Method of Determination Total Protein Content

After treatment, ten treated adult insects (five males and five females) were ground into a fine powder, and an accurate 0.1 g sample was weighed and placed into a centrifuge tube. A 0.9 mL volume of PBS solution was added to the centrifuge tube, mixed well, and centrifuged at 2500 rpm for 10 min; then, the supernatant was collected. The supernatant was mixed with physiological saline at a ratio of 1:19, and 10 μL of the diluted mixture was taken. The sample was added to the reaction system configured as shown in Table S2, and the absorbance (OD value) was measured at a wavelength of 562 nm.

### 2.6. Activity Assay Method

#### 2.6.1. Superoxide Dismutase Activity Assay Method

Ten treated adult insects (five males and five females) from each treatment were ground into a fine powder. Exactly 0.1 g of the powder was accurately weighed using a balance and placed into a centrifuge tube. Then, 0.9 mL of PBS solution was added to the tube, mixed thoroughly, and centrifuged at 8000 rpm for 10 min. The supernatant was collected as the sample. The absorbance (OD value) of the relative reaction system was measured at a wavelength of 560 nm (Table S3).

#### 2.6.2. Peroxidase Activity Assay Method

Ten treated adult insects (five males and five females) from each treatment were ground into a fine powder. Exactly 0.1 g of the powder was accurately weighed using a balance and placed into a centrifuge tube. Then, 0.9 mL of PBS solution was added to the tube, mixed thoroughly, and centrifuged at 5000 rpm for 10 min. The supernatant was collected as the sample. The absorbance (OD value) of the relative reaction system was measured at a wavelength of 450 nm (Table S4).

#### 2.6.3. Catalase Activity Assay Method

Ten treated adult insects (five males and five females) from each treatment were ground into a fine powder. Exactly 0.1 g of the powder was accurately weighed using a balance and placed into a centrifuge tube. Then, 0.9 mL of PBS solution was added to the tube, mixed thoroughly, and centrifuged at 4000 rpm for 10 min. The supernatant was collected as the sample. The absorbance (OD value) of the relative reaction system was measured at a wavelength of 405 nm (Table S5).

#### 2.6.4. Total Antioxidant Capacity Assay Method

Ten treated adult insects (five males and five females) from each treatment were ground into a fine powder. Exactly 0.02 g of the powder was accurately weighed using a balance and placed into a centrifuge tube. Then, 100 μL of PBS solution was added to the tube, mixed thoroughly, and centrifuged at 12,000 rpm for 10 min. The supernatant was collected as the sample. The absorbance (OD value) of the relative sample was measured at a wavelength of 414 nm.

#### 2.6.5. Malondialdehyde Activity Assay Method

Ten treated adult insects (five males and five females) from each treatment were ground into a fine powder. Exactly 0.1 g of the powder was accurately weighed using a balance and placed into a centrifuge tube. Then, 0.9 mL of PBS solution was added to the tube, mixed thoroughly, and centrifuged at 10,000 rpm for 10 min. The supernatant was collected as the sample. The absorbance (OD value) of the relative reaction system was measured at a wavelength of 532 nm (Table S6).

### 2.7. Data Analysis

Data were processed and analyzed using SPSS 26 statistical software (International Business Machines Corporation, Armonk, NY, USA). One-way analysis of variance (ANOVA) was used to analyze the significant differences in the antioxidant enzyme activities of adult insects under different treatment conditions (*p* < 0.05). Two-way ANOVA was performed with temperature and exposure duration as fixed factors. Data were tested for normality and homogeneity of variance prior to ANOVA (Supplementary Tables S7–S16). Duncan’s new multiple range test was used for multiple comparisons to test for significant differences. Independent-sample t-tests were conducted to analyze the differences in energy substances between female and male adults under the same treatment conditions (*p* < 0.05).

## 3. Results

### 3.1. Effects of Short-Term High-Temperature Stress on Energy Substances in Adult R. pedestris

#### 3.1.1. Effects of Short-Term High-Temperature Stress on Water Loss Rate of *R. pedestris* Adults

Temperature and exposure time exhibited significant effects on the water loss rate in both sexes (♀: *F* = 10.56; *p* < 0.001; ♂: *F* = 4.84; *p* < 0.001). The effect of temperature depends on the exposure duration. Temperature significantly affected body water loss: at each exposure duration, the water loss rate increased with rising temperature. After 1 h, the water loss rates at 24–32 °C were significantly lower than those at 36–44 °C (females: *F* = 16.94, *p* < 0.001; males: *F* = 14.61, *p* < 0.001). The maximum rates occurred at 44 °C for both sexes (females: 6.89 ± 2.34%; males: 7.89 ± 2.53%), with no significant intersexual difference. After 2 h, the water loss rates at 24–36 °C were significantly lower than at 40–44 °C (females: *F* = 36.80, *p* < 0.001; males: *F* = 50.60, *p* < 0.001). The peak values were observed at 44 °C (females: 19.47 ± 4.86%; males: 18.12 ± 4.17%), with no significant difference between sexes. After 3 h, the water loss rates at 24 °C were significantly lower than at 32–44 °C (females: *F* = 44.49, *p* < 0.001; males: *F* = 45.88, *p* < 0.001). The maximum rates occurred at 44 °C (females: 18.83 ± 3.27%; males: 21.34 ± 7.24%), with no significant intersexual difference. After 4 h, the water loss rates at 24 °C were significantly lower than at 32–44 °C (females: *F* = 33.79, *p* < 0.001; males: *F* = 12.06, *p* < 0.001). The peak values were observed at 44 °C (females: 26.97 ± 12.75%; males: 24.43 ± 20.07%), with no significant difference between sexes.

**Figure 1:**
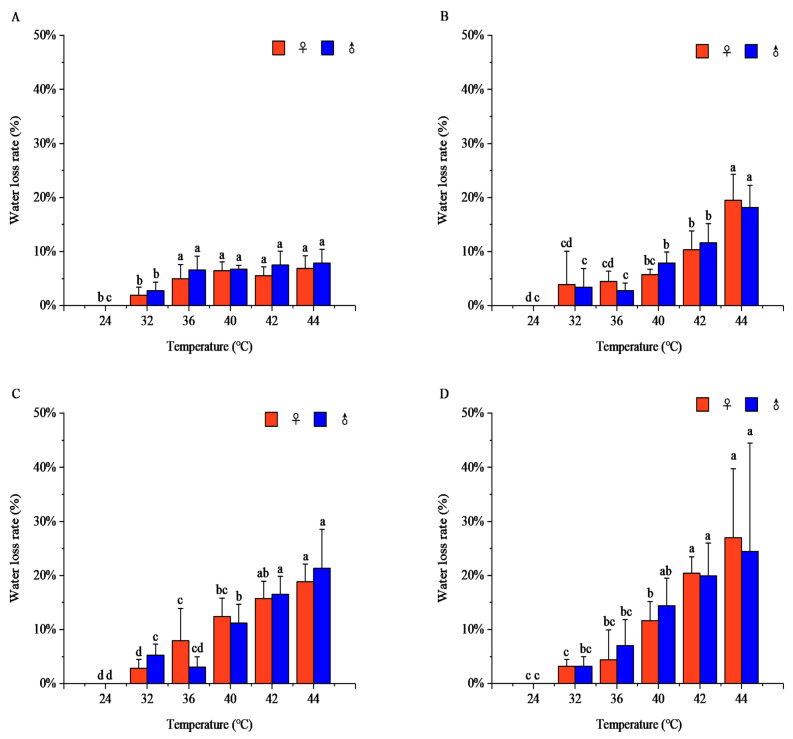
Water loss rate of *Riptortus pedestris* under different temperature and time treatments. Note: Lower letters indicate differences at the same time under different temperatures of the same gender (*p* < 0.05). (**A**) 1 h of heat stress; (**B**) 2 h of heat stress; (**C**) 3 h of heat stress; (**D**) 4 h of heat stress.

At 32 °C, the water loss rate increased initially and then stabilized with prolonged exposure (females: *F* = 0.92, *p* = 0.44; males: *F* = 2.71, *p* = 0.06). At 36 °C, females exhibited an increase–decrease–increase pattern (*F* = 2.21, *p* = 0.09), peaking at 3 h (7.96 ± 5.96%), whereas males showed a decrease–increase pattern, reaching a maximum at 4 h (7.06 ± 4.76%; *F* = 7.83, *p* < 0.001). At 40 °C, the water loss rate increased gradually with exposure duration (females: *F* = 25.04, *p* < 0.001; males: *F* = 16.39, *p* < 0.001); the rates at 1–2 h were significantly lower than at 3–4 h, with female and male peaks at 3 h (12.45 ± 3.33%) and 4 h (14.43 ± 5.06%), respectively. At 42 °C and 44 °C, the water loss rates increased significantly with exposure time (42 °C females: *F* = 69.82, *p* < 0.001; males: *F* = 26.45, *p* < 0.001; 44 °C females: *F* = 17.99, *p* < 0.001; males: *F* = 5.87, *p* < 0.001). At 42 °C, the maximum rates at 4 h were 20.39 ± 3.07% (females) and 19.93 ± 6.04% (males); at 44 °C, the maximum rates at 4 h were 26.97 ± 12.75% (females) and 24.43 ± 20.07% (males) (Figure 1).

Within the suitable temperature range (24–32 °C), exposure time had no significant effect on the adult water loss rate; however, under sublethal (36–40 °C) and lethal high temperatures (42–44 °C), the water loss rate increased significantly with prolonged exposure (Table S7). This pattern is consistent with the reduced stability of water homeostasis under extreme thermal stress in *R. pedestris* adults.

#### 3.1.2. Effects of Short-Term High-Temperature Stress on Fat Content of Adult *R. pedestris*

Temperature and exposure duration showed significant effects on fat content in both females and males (females: *F* = 10.25, *p* < 0.001; males: *F* = 14.65, *p* < 0.001). Overall, fat content displayed nonlinear responses to increasing temperature and prolonged stress, with distinct sexual differences under specific treatments.

**Figure 2:**
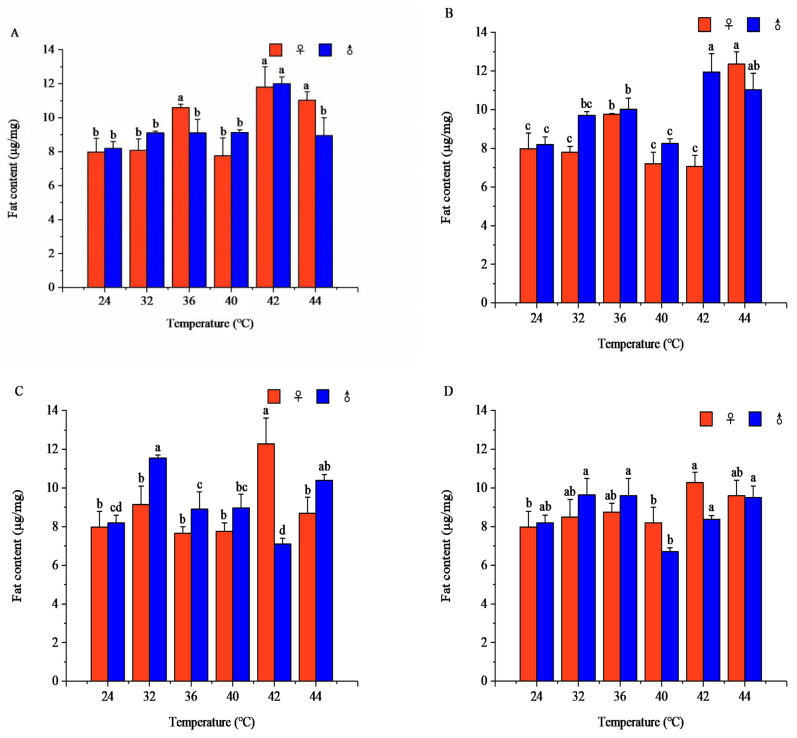
Fat content of *Riptortus pedestris* under different temperature and time treatments. Note: Lower letters indicate differences at the same time under different temperatures of the same gender (*p* < 0.05). (**A**) 1 h of heat stress; (**B**) 2 h of heat stress; (**C**) 3 h of heat stress; (**D**) 4 h of heat stress.

Under most durations, fat content shifted between increasing and decreasing patterns as temperature rose. Females generally reached fat peaks under moderate–high temperatures at medium exposure times, whereas males often attained maximal values at shorter durations (Figure 2). Detailed variation patterns, peak values, and statistical comparisons are provided in Table S8.

#### 3.1.3. Effects of Short-Term High-Temperature Stress on Total Sugar Content of Adult *R. pedestris*

**Figure 3:**
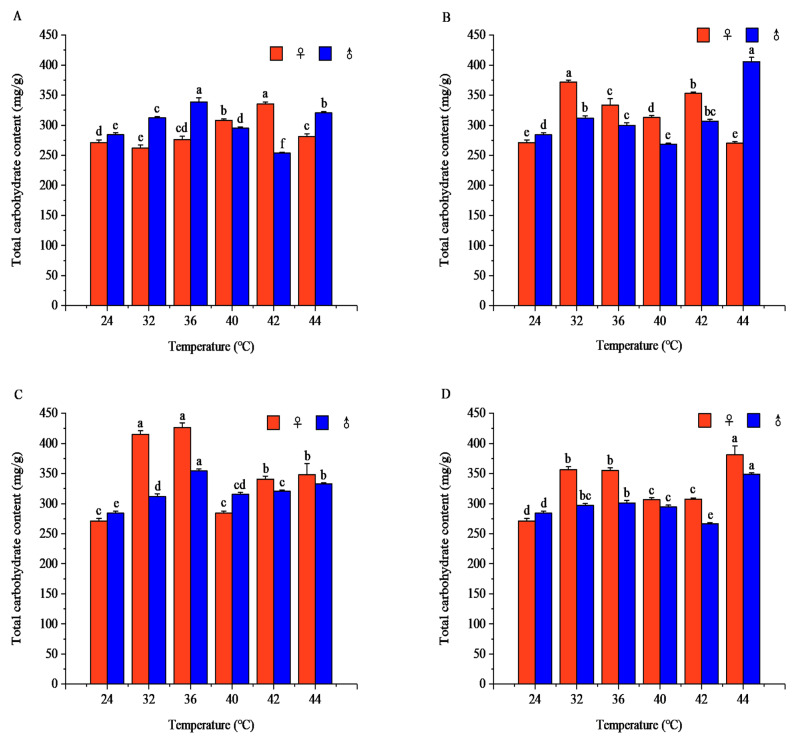
Total carbohydrate contents of *Riptortus pedestris* under different temperature and time treatments. Note: Lower letters indicate differences at the same time under different temperatures of the same gender (*p* < 0.05). (**A**) 1 h of heat stress; (**B**) 2 h of heat stress; (**C**) 3 h of heat stress; (**D**) 4 h of heat stress.

The effects of temperature and exposure duration on total sugar content were significant in both sexes. The total sugar content generally responded to rising temperature with increases followed by decreases or continuous elevation, and marked sexual differences were detected under all treatments (*p* < 0.001). The sugar dynamics over exposure time also differed between sexes, with peak timing shifting with temperature intensity (Figure 3). Full descriptive patterns, peak concentrations, and statistical results are summarized in Table S9.

#### 3.1.4. Effects of Short-Term High-Temperature Stress on Glycogen Content of Adult *R. pedestris*

**Figure 4:**
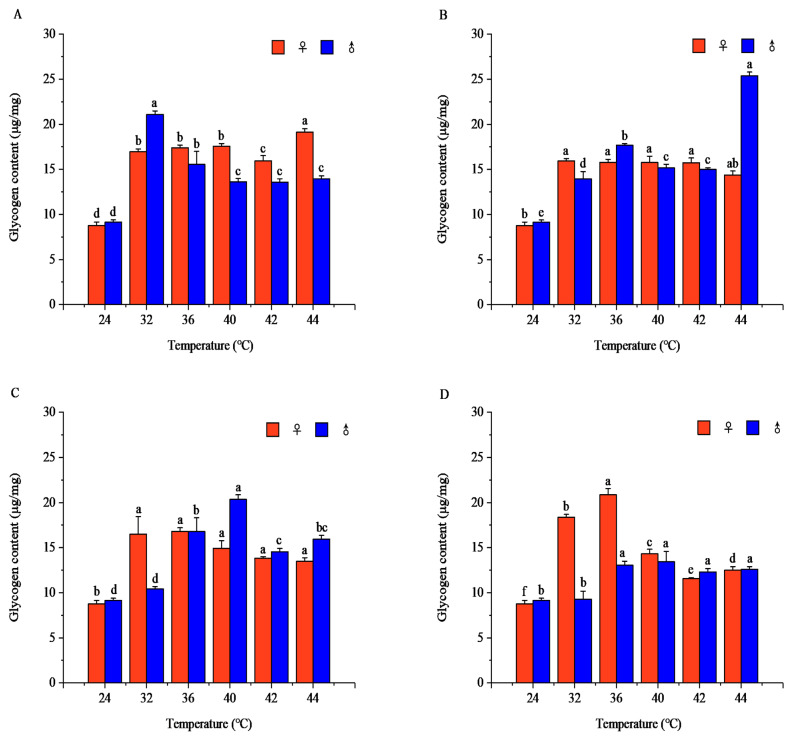
Glycogen contents of *Riptortus pedestris* under different temperature and time treatments. Note: Lower letters indicate differences at the same time under different temperatures of the same gender (*p* < 0.05). (**A**) 1 h of heat stress; (**B**) 2 h of heat stress; (**C**) 3 h of heat stress; (**D**) 4 h of heat stress.

Temperature and exposure duration showed significant effects on glycogen content in both sexes (*p* < 0.001). Glycogen levels displayed nonlinear, temperature- and duration-dependent fluctuations and exhibited strong sexual differences under nearly all treatments. Females and males reached peak glycogen contents under distinct temperature–time combinations, with divergent response trajectories across stress intensities (Figure 4). Detailed patterns, peak values, and statistical comparisons are provided in Table S10.

#### 3.1.5. Effects of Short-Term High-Temperature Stress on Total Protein Content of Adult *R. pedestris*

**Figure 5:**
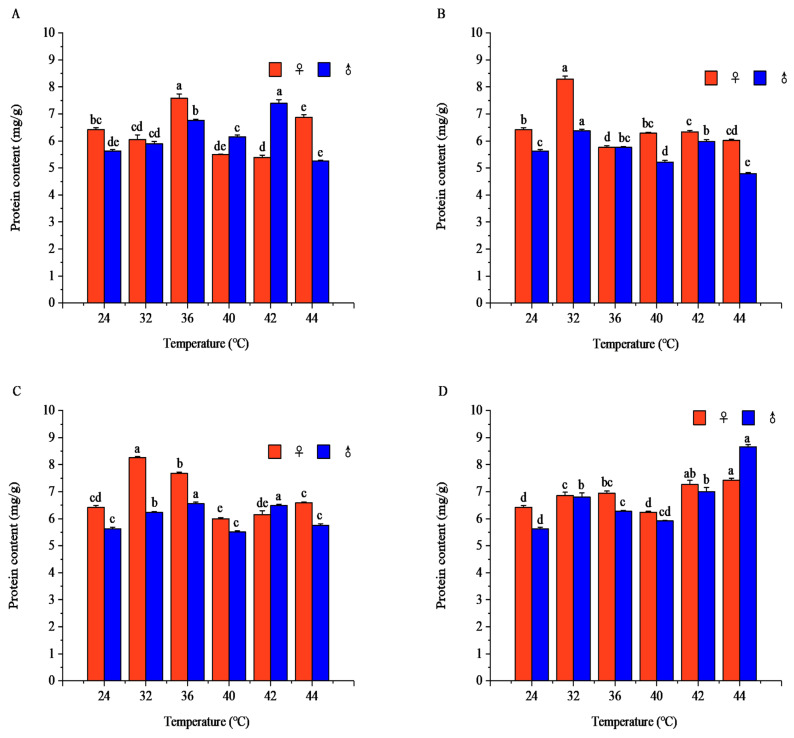
Protein contents of *Riptortus pedestris* under different temperature and time treatments. Note: Lower letters indicate differences at the same time under different temperatures of the same gender (*p* < 0.05). (**A**) 1 h of heat stress; (**B**) 2 h of heat stress; (**C**) 3 h of heat stress; (**D**) 4 h of heat stress.

Temperature and exposure duration showed significant effects on total protein content in both sexes (*p* < 0.001). Protein content displayed nonlinear, temperature- and duration-dependent responses and showed marked sexual differences under most treatments. Overall, protein levels fluctuated with increasing temperature, showing rising–falling patterns or gradual elevation across exposure times. The peak values and response trajectories differed considerably between females and males, with peak timing and temperature shifting with treatment intensity (Figure 5). Detailed response patterns, peak concentrations, statistical values, and pairwise comparisons are provided in Table S11.

### 3.2. Effects of Short-Term High-Temperature Stress on Antioxidant Enzyme Activities of Adult R. pedestris

#### 3.2.1. Effects of Short-Term Heat Stress on SOD Activity in Adult *R. pedestris*

Temperature and exposure time significantly affected SOD activity in both sexes, with the temperature effect dependent on exposure duration.

After 1 h of exposure, the SOD activity in both sexes showed an up–down–up–down trend with increasing temperature (females: *F* = 11.705, *p* < 0.001; males: *F* = 17.352, *p* < 0.001). The female peak was at 36 °C (4.61 ± 0.72 U/mg), and the male peak at 32 °C (5.53 ± 0.36 U/mg), with significant sexual differences at 24, 32, 40, and 44 °C.

After 2 h of exposure, both sexes exhibited the same up–down–up–down pattern (females: *F* = 177.454, *p* < 0.001; males: *F* = 6.188, *p* < 0.001). Females peaked at 32 °C (5.25 ± 0.36 U/mg), and males at 42 °C (5.87 ± 0.10 U/mg); significant sexual differences occurred at 24, 36, 42, and 44 °C.

**Figure 6:**
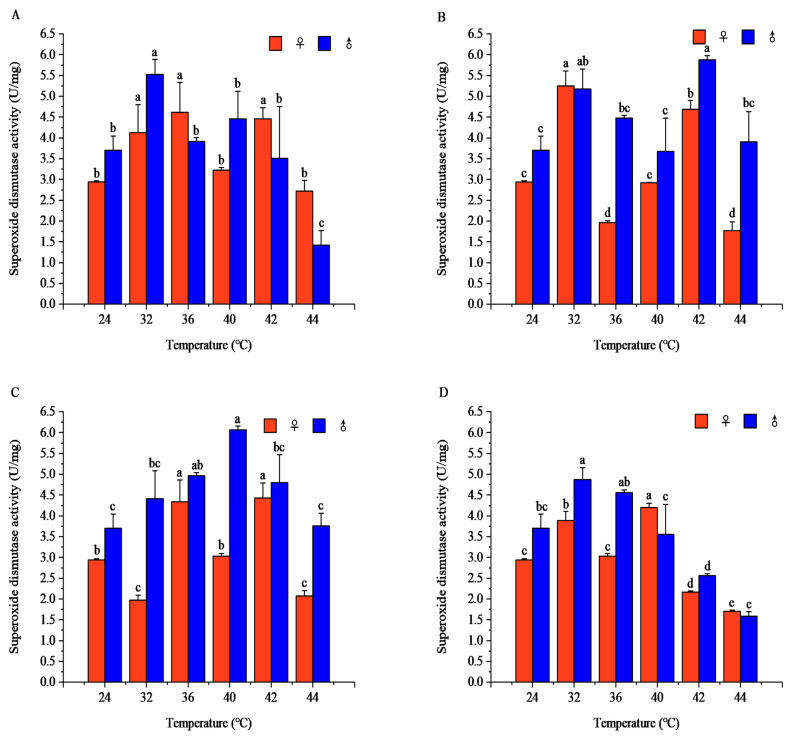
Superoxide dismutase (SOD) activity of *Riptortus pedestris* under different temperature and time treatments. Note: Lower letters indicate differences at the same time under different temperatures of the same gender (*p* < 0.05). (**A**) 1 h of heat stress; (**B**) 2 h of heat stress; (**C**) 3 h of heat stress; (**D**) 4 h of heat stress.

After 3 h and 4 h of exposure, males followed an up–down–up–down trend, while females displayed a simple up–down pattern (all *p* < 0.001). At 3 h of exposure, females peaked at 42 °C and males at 40 °C; at 4 h of exposure, the female peak was at 40 °C and the male peak at 44 °C. Significant sexual differences existed at specific temperature points at both 3 h and 4 h of exposure (Figure 6).

At constant temperatures, the SOD activity varied distinctly with exposure time between sexes. At 32 °C, females showed an up–down–up trend and males a down–up trend, both peaking at 2 h of exposure. At 36 °C, females peaked at 1 h of exposure and males at 3 h of exposure; at 40 °C, the female maximum appeared at 4 h of exposure and the male maximum at 3 h of exposure. At 42 °C, the SOD activity in both sexes rose first and then fell and peaked at 2 h of exposure. At 44 °C, female activity declined continuously with exposure time and peaked at 1 h of exposure, whereas males showed an up–down pattern with a peak at 2 h of exposure (Table S12).

#### 3.2.2. Effects of Short-Term High-Temperature Stress on POD Activity in Adult *R. pedestris*

Temperature and exposure time significantly affected POD activity in both sexes, and the temperature effect relied on exposure duration.

After 1 h of exposure, the POD activity at 24 °C and 44 °C was markedly higher than at 32–42 °C (females: *F* = 64.79, *p* < 0.001; males: *F* = 32.41, *p* < 0.001). Females peaked at 44 °C (4.43 ± 0.18 U/mg), and males at 24 °C (4.37 ± 0.10 U/mg), with significant sexual differences at 32, 36, 40, 42, and 44 °C.

After 2 h of exposure, the higher POD activity at 24 °C and 44 °C relative to 32–42 °C remained significant (females: *F* = 25.53, *p* < 0.001; males: *F* = 43.31, *p* < 0.001). Both sexes reached their peaks at 24 °C, and significant sexual differences occurred at 32, 36, 40, and 42 °C.

After 3 h of exposure, the POD activity still maintained higher levels at 24 °C and 44 °C (females: *F* = 52.18, *p* < 0.001; males: *F* = 51.74, *p* < 0.001). The female peak was at 44 °C, and the male peak at 24 °C; obvious sexual differences were observed at 32, 36, 42, and 44 °C.

**Figure 7:**
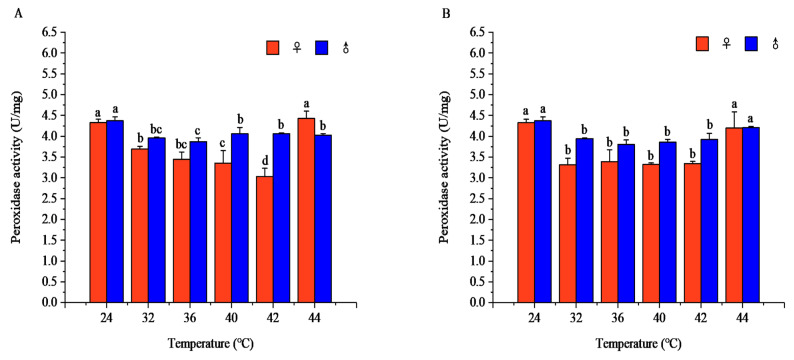

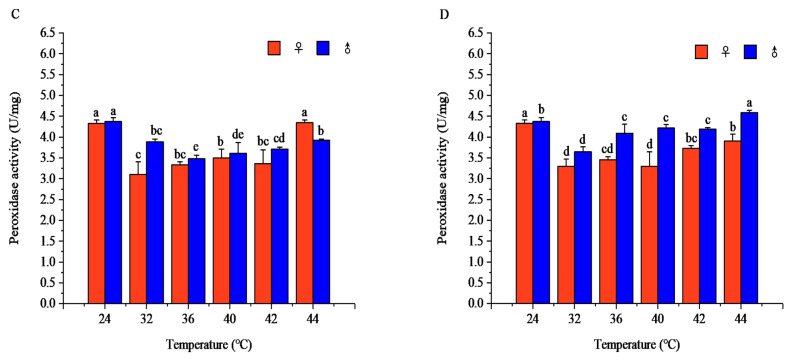
Peroxidase (POD) activity of *Riptortus pedestris* under different temperature and time treatments. Note: Lower letters indicate differences at the same time under different temperatures of the same gender (*p* < 0.05). (**A**) 1 h of heat stress; (**B**) 2 h of heat stress; (**C**) 3 h of heat stress; (**D**) 4 h of heat stress.

After 4 h of exposure, the same high-activity pattern at 24 °C and 44 °C was observed (females: *F* = 32.77, *p* < 0.001; males: *F* = 45.30, *p* < 0.001). Females peaked at 24 °C, while males peaked at 44 °C, with significant sexual differences detected at all the tested temperatures (Figure 7).

At each exposure duration, POD activity presented a decrease–increase trend with rising temperature, being consistently higher at 24 °C and 44 °C. At 32 °C, the POD activity in both sexes gradually declined with prolonged exposure and peaked at 1 h. At 36 °C, females showed no significant temporal variation, whereas males followed a decrease–increase pattern and peaked at 4 h. At 40 °C and 42 °C, females had non-significant fluctuating trends, while males displayed a decrease–increase pattern with peak activity at 4 h. At 44 °C, female POD activity decreased gradually and maximized at 1 h, whereas males showed an increase–decrease pattern and peaked at 4 h (Table S13).

#### 3.2.3. Effects of Short-Term High-Temperature Stress on the CAT Activity of Adult *R. pedestris* with Increasing Temperature

Temperature and exposure time significantly affected CAT activity in both sexes, with the temperature effect dependent on exposure duration.

After 1 h of exposure, female CAT activity rose with increasing temperature, while males presented an initial decrease followed by a rise (females: *F* = 184.76, *p* < 0.001; males: *F* = 2083.85, *p* < 0.001). Females peaked at 44 °C, and males at 24 °C, with significant sexual differences across all temperatures.

After 2 h of exposure, females still showed rising CAT activity with temperature, whereas males decreased first then increased (females: *F* = 476.32, *p* < 0.001; males: *F* = 7.97, *p* = 0.002). The female peak appeared at 40 °C, and the male peak remained at 24 °C; significant sexual differences existed at all tested temperatures.

After 3 h of exposure, CAT activity in both sexes declined in a fluctuating manner as temperature increased (females: *F* = 932.42, *p* < 0.001; males: *F* = 24.42, *p* < 0.001). Females peaked at 40 °C, and males at 24 °C, with significant sexual differences at all temperature points.

**Figure 8:**
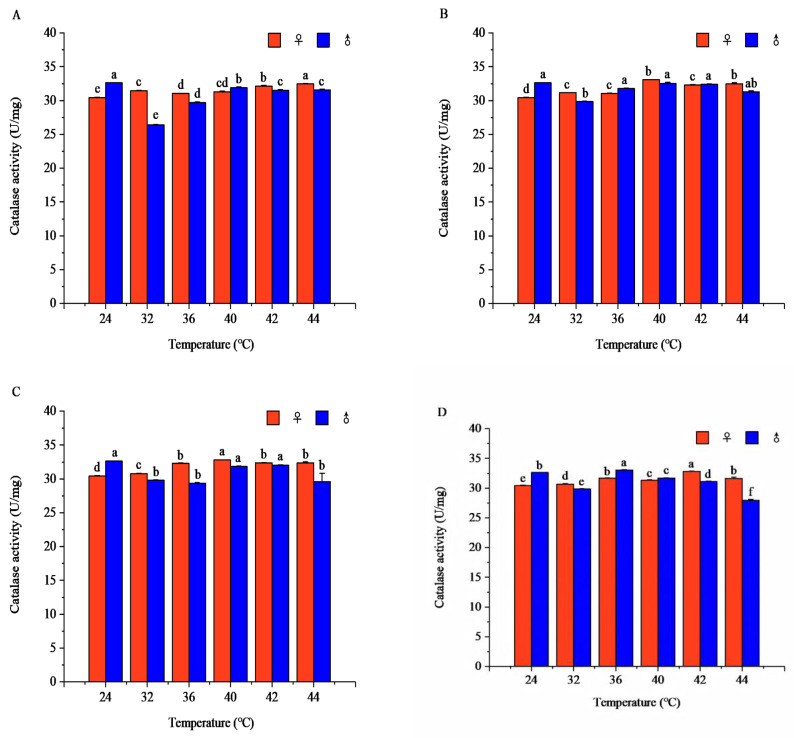
Catalase (CAT) activity of *Riptortus pedestris* under different temperature and time treatments. Note: Lower letters indicate differences at the same time under different temperatures of the same gender (*p* < 0.05). (**A**) 1 h of heat stress; (**B**) 2 h of heat stress; (**C**) 3 h of heat stress; (**D**) 4 h of heat stress.

After 4 h of exposure, CAT activity followed an increase–decrease trend with rising temperature (females: *F* = 156.07, *p* < 0.001; males: *F* = 1452.25, *p* < 0.001). The female peak was at 42 °C, the male peak shifted to 36 °C, and sexual differences were significant at all temperatures (Figure 8).

At fixed temperatures, CAT activity changed distinctly with exposure time between sexes. At 32 °C, female activity declined gradually and was maximized at 1 h, while males increased steadily and peaked at 4 h. At 36 °C, both sexes showed a fluctuating increasing trend, with females peaking at 3 h and males at 4 h. At 40 °C, CAT activity in both sexes increased first then decreased and reached its maximum at 2 h, while males had no significant temporal change. At 42 °C, females increased gradually to a maximum at 4 h, whereas males followed an increase–decrease pattern and peaked at 2 h. At 44 °C, females presented an increase–decrease trend with a peak at 2 h, while males declined continuously and peaked at 1 h (Table S14).

#### 3.2.4. Effects of Short-Term High-Temperature Stress on T-AOC Capacity of Adult *R. pedestris*

Temperature and exposure time significantly affected T-AOC in both sexes, and the temperature effect relied on exposure duration.

After 1 h of exposure, T-AOC in females followed a decrease–increase–decrease pattern, while males declined gradually with rising temperature (females: *F* = 29.73, *p* < 0.001; males: *F* = 10.82, *p* < 0.001). Females peaked at 42 °C, and males at 24 °C, with significant sexual differences at 36, 40, and 42 °C.

After 2 h of exposure, females showed a decrease–increase trend, whereas males displayed an increase–decrease–increase pattern (females: *F* = 32.53, *p* < 0.001; males: *F* = 67.03, *p* < 0.001). The female maximum occurred at 44 °C, and the male peak at 32 °C; significant sexual differences were found at all detected temperatures.

After 3 h of exposure, females maintained a decrease–increase pattern, while males presented an increase–decrease trend (females: *F* = 19.11, *p* < 0.001; males: *F* = 56.20, *p* < 0.001). Females peaked at 42 °C, and males at 32 °C, with obvious sexual differences across most temperature points.

**Figure 9:**
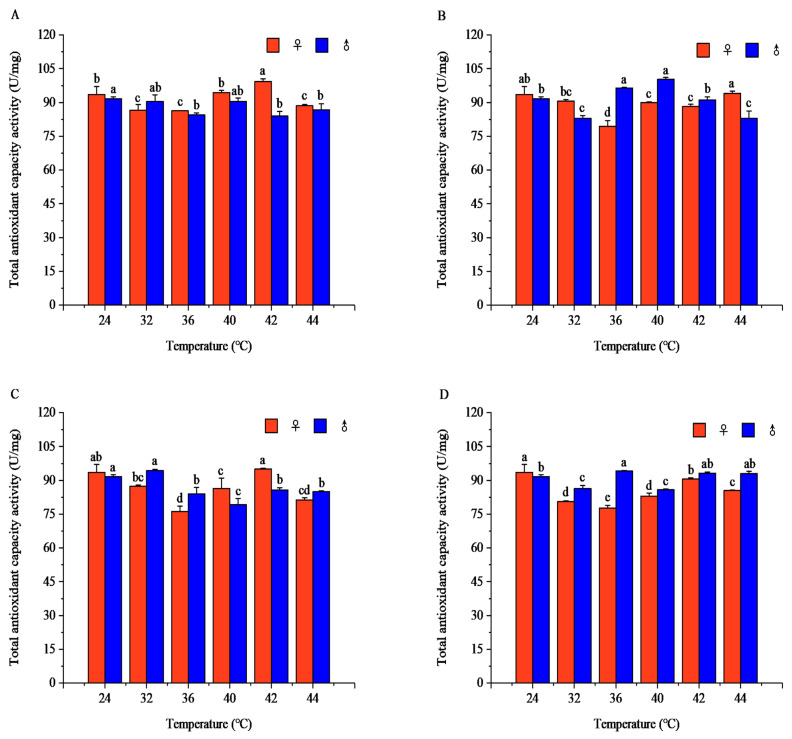
Total antioxidant capacity (T-AOC) of *Riptortus pedestris* under different temperature and time treatments. Note: Lower letters indicate differences at the same time under different temperatures of the same gender (*p* < 0.05). (**A**) 1 h of heat stress; (**B**) 2 h of heat stress; (**C**) 3 h of heat stress; (**D**) 4 h of heat stress.

After 4 h of exposure, females still showed a decrease–increase pattern, whereas males increased gradually with temperature elevation (females: *F* = 53.39, *p* < 0.001; males: *F* = 80.74, *p* < 0.001). Females reached the highest value at 24 °C, and males at 36 °C, with significant sexual differences at all tested temperatures (Figure 9).

At fixed temperatures, T-AOC varied distinctly with exposure time between sexes. At 32 °C, females followed an increase–decrease trend and peaked at 2 h, while males showed a decrease–increase–decrease pattern with a maximum at 3 h. At 36 °C and 40 °C, female T-AOC decreased gradually and peaked at 1 h, whereas males exhibited a decrease–increase–decrease pattern and reached the highest level at 2 h. At 42 °C, females showed a fluctuating trend with a peak at 1 h, while males increased steadily and maximized at 4 h. At 44 °C, females presented a decrease–increase–decrease pattern peaking at 2 h, and males followed a decrease–increase trend with a maximum at 4 h (Table S15).

#### 3.2.5. Effects of Short-Term High-Temperature Stress on MDA Content of Adult *R. pedestris*

Temperature and exposure time significantly affected MDA content in both sexes, with the temperature effect dependent on exposure duration.

After 1 h of exposure, the MDA content in both sexes displayed an increase–decrease trend with rising temperature (females: *F* = 10.41, *p* < 0.001; males: *F* = 4.66, *p* = 0.009). Both sexes peaked at 32 °C, and significant sexual differences occurred at 24, 32, 36, and 42 °C.

After 2 h of exposure, MDA still followed an increase–decrease pattern (females: *F* = 12.00, *p* < 0.001; males: *F* = 3.90, *p* = 0.014), with maximum values in both sexes at 32 °C. Significant sexual differences were detected at 24, 42, and 44 °C.

After 3 h of exposure, the increase–decrease trend remained consistent (females: *F* = 15.98, *p* < 0.001; males: *F* = 10.57, *p* < 0.001). Females peaked at 32 °C while males peaked at 36 °C, with obvious sexual differences at 24, 32, 36, and 44 °C.

**Figure 10:**
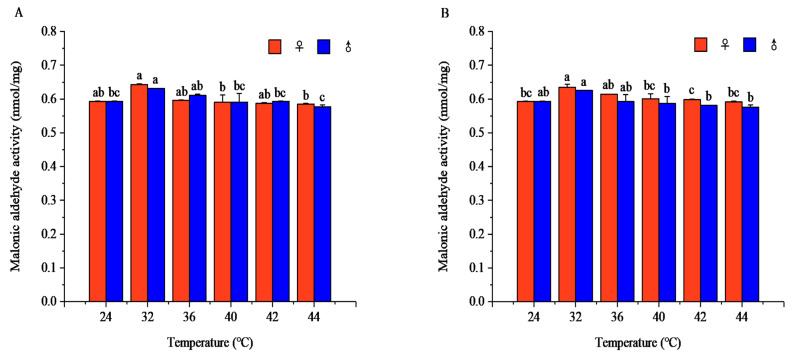

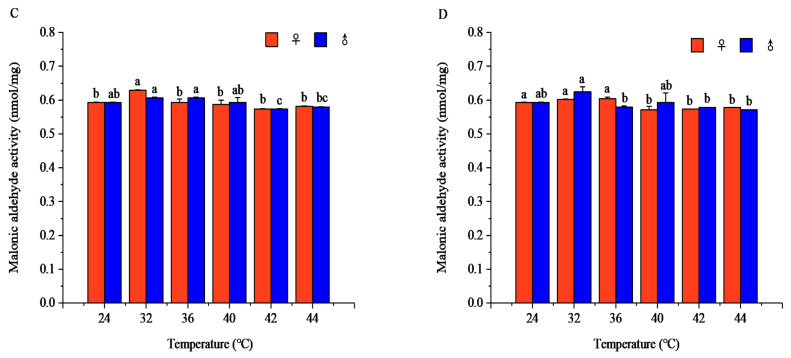
Malonic aldehyde (MDA) activity of *Riptortus pedestris* under different temperature and time treatments. Note: Lower letters indicate differences at the same time under different temperatures in the same gender (*p* < 0.05). (**A**) 1 h of heat stress; (**B**) 2 h of heat stress; (**C**) 3 h of heat stress; (**D**) 4 h of heat stress.

After 4 h of exposure, MDA content maintained the increase–decrease pattern (females: *F* = 24.56, *p* < 0.001; males: *F* = 4.75, *p* = 0.006). The female peak shifted to 36 °C, and the male peak remained at 32 °C; significant sexual differences were observed at 24, 36, 42, and 44 °C (Figure 10).

At fixed temperatures, MDA content changed distinctly with exposure time. At 32 °C and 42 °C, MDA content in both sexes declined over time and reached the highest level at 1 h. At 36 °C, both sexes showed a continuous decreasing trend, with females peaking at 2 h and males at 1 h. At 40 °C, females presented an increase–decrease pattern peaking at 2 h, while males had a non-significant fluctuating trend. At 44 °C, females followed an increase–decrease pattern with a maximum at 2 h, whereas males showed an insignificant decrease–increase–decrease trend and peaked at 3 h (Table S16).

## 4. Discussion

This study investigated the effects of short-term high-temperature stress on the energy substances and antioxidant enzyme activities of adult *R. pedestris*. It provides a theoretical basis for the prediction, forecasting, and integrated pest management of this species, and lays a foundation for clarifying the impact of temperature on the population dynamics of *R. pedestris* under global climate warming.

Short-term high-temperature stress significantly affects insect water balance regulation and the accumulation and metabolism of energy substances [29,30]. Under high-temperature stress, water evaporation in insects accelerates, leading to severe water loss from the body and body surface. Within the sublethal high-temperature range, the water loss rate of *R. pedestris* increased with rising temperature and prolonged exposure time. At 44 °C for 4 h, the water loss rates of female and male adults reached 26.97% ± 12.75% and 24.43% ± 20.07%, respectively, which were significantly higher than those in the suitable temperature and sublethal high-temperature ranges. Within the lethal high-temperature range, the water loss rate increased significantly [31]. For example, the water loss rate of *Romalea guttata* (lubber grasshopper) was significantly higher at 43 °C than at other temperatures, and the water loss rate of *Diceroprocta apache* (cactus dodger cicada) increased rapidly at 45.5 °C, which is consistent with our results [32,33]. When the temperature exceeds a critical threshold, the insect’s water loss rate increases sharply [34]. The absence of significant sexual differences in water loss rates suggests that males and females employ comparable water conservation strategies or face similar desiccation risks under the tested conditions, though underlying mechanisms may differ.

Fat is an important long-term energy storage substance in insects. Under high-temperature stress, insects convert fat into fatty acids, glycerol, and other substances to meet energy demands induced by high temperatures [35]. In *R. pedestris*, lipid content was significantly higher under sublethal and lethal high-temperature ranges than in the optimal temperature zone and showed a fluctuating increasing trend with rising temperature and prolonged stress duration, with females consistently maintaining higher lipid levels than males. The observed increase in lipid content under heat stress may reflect a shift toward energy storage rather than immediate catabolism. However, whether this represents an adaptive strategy or a metabolic bottleneck remains unclear. The potential role of lipids in maintaining membrane fluidity would depend on changes in fatty acid composition [36], which were not assessed in this study. On one hand, lipolysis releases fatty acids and glycerol to support energy-intensive stress responses, including the synthesis of heat shock proteins, maintenance of ion homeostasis, and activation of antioxidant systems. On the other hand, lipids help preserve cell membrane fluidity under thermal stress and serve as a stabilizing buffer that reduces the risk of membrane damage, thereby enhancing heat tolerance [8]. Studies on *Bactrocera dorsalis* and *Chelinidea vittiger* have also shown that females have higher fat contents than males, which may be due to fat accumulation in reproductive organs such as ovaries [17,35,36,37]. *R. pedestris* displayed an unusual strategy compared with some other insects such as *Megacopta cribraria*, in which lipid content declines under warm conditions. Instead of decreasing, lipid levels increased under short-term high-temperature stress, implying that *R. pedestris* prioritizes lipid anabolism and storage over immediate catabolism under extreme heat. This strategy may be adaptive: under heat-induced water loss and metabolic disturbance, lipids serve as a stable, osmotically neutral energy source that avoids the large osmotic fluctuations caused by extensive carbohydrate catabolism, thereby supporting survival under acute thermal stress.

Carbohydrates are another important energy reserve in insects. High-temperature stress promotes the decomposition of glycogen into glucose and other monosaccharides (including glucose, fructose, and other sugars), which are rapid energy sources [38]. Under short-term high-temperature stress, *R. pedestris* synthesizes more carbohydrates to enhance energy reserves and improve heat tolerance. Glycogen and total sugar contents were generally higher in the sublethal and lethal high-temperature ranges than in the suitable temperature range. Under short-term high-temperature stress, insect metabolism accelerates, so glycogen and total sugar are preferentially consumed to meet energy demands for adapting to environmental changes [20]. However, some studies have shown that insects can switch their energy utilization patterns to adapt to environmental changes [39]. For example, *Pieris rapae* larvae showed an initial increase followed by a decrease in total sugar content under 28–42 °C high-temperature stress [14], which is consistent with the carbohydrate metabolism trend of *R. pedestris* in the high-temperature range. Research indicates that *R. pedestris* is a carbohydrate-utilizing species within the 16–36 °C range, with carbohydrates being the main metabolic energy source [25].

Total protein is a critical component of insect cellular function and metabolism [30]. Within the 24–44 °C range, at exposure times of 1–3 h, the total protein content of *R. pedestris* first increased and then decreased, with lower content in the lethal high-temperature range than in the suitable temperature range. At 4 h of exposure, total protein content increased, with higher content in the lethal high-temperature range than in the sublethal high-temperature range. For example, *Oryzaephilus surinamensis* (sawtoothed grain beetle) showed significantly reduced protein content in the 42–45 °C lethal high-temperature range [40], and *Tetranychus truncatus* (carmine spider mite) had significantly decreased protein content at 42 °C [41], which is consistent with our findings that *R. pedestris* has lower total protein content in the lethal high-temperature range than in the suitable temperature range. In contrast, *Tribolium castaneum* (red flour beetle) showed increased protein content at 45 °C [42], which may be due to differences in stress response mechanisms, heat shock protein synthesis capacity, or cellular damage sensitivity among species. Insects synthesize heat shock proteins, antioxidant enzymes, and other stress-related proteins to adapt to temperature changes and improve heat tolerance [30]. Short-term high temperatures accelerate cellular metabolism, promoting protein degradation and heat shock protein synthesis to provide energy and enhance heat tolerance for adapting to high-temperature environments [30]. Initially, insects adapt to high temperatures by synthesizing heat shock proteins and degrading damaged proteins; however, as temperatures rise to the lethal high-temperature range, oxidative damage impairs cellular and tissue metabolic function, leading to a decrease in total protein content [30,40,41], which aligns with the total protein content trend of *R. pedestris* under high-temperature stress.

The complex patterns of fat, total sugar, glycogen, and protein contents observed in this study reflect the sophisticated energy reallocation strategies employed by *R. pedestris* to cope with thermal stress. The initial increase in energy reserves at moderate high temperatures followed by depletion at extreme temperatures suggests a biphasic response: energy mobilization to fuel protective mechanisms at sublethal temperatures, and metabolic exhaustion or disruption at lethal temperatures. Notably, sexual differences in energy substrate dynamics were frequently observed, with females and males often peaking at different temperatures and time points. For instance, the females’ total sugar content peaked at 42 °C after 1 h, while the males’ peaked at 36 °C; glycogen content showed even more pronounced sex-specific patterns. These differences may be attributed to divergent reproductive strategies, body size differences, or sex-specific thermal sensitivities. The fluctuating patterns of protein content suggest active protein synthesis and degradation processes, possibly involving heat shock protein production and metabolic enzyme turnover.

In summary, the changes in energy substances under short-term high-temperature stress in *R. pedestris* do not simply reflect ‘enhanced reserves’ or ‘accelerated consumption’. These changes may reflect a reallocation of energy substrates, though the specific functional roles (e.g., osmoregulation, membrane stabilization) remain speculative without direct measurements of membrane properties or metabolite fluxes. This strategy is partially adaptive under sublethal high temperatures, but under lethal high temperatures, energy metabolite pathways become constrained by both ROS damage and water imbalance, ultimately leading to physiological collapse.

Short-term high-temperature stress induces insects to develop mechanisms for adapting to temperature changes, and alterations in antioxidant enzyme activity are a key physiological response to temperature fluctuations [43]. High-temperature stress causes oxidative damage in insects, triggering an oxidative stress response [44]. To alleviate and eliminate intracellular ROS and prevent damage to lipids, proteins, and DNA, insects possess an antioxidant defense system consisting of enzymes including SOD, CAT, and POD, which help scavenge excess ROS [40,43]. In the sublethal high-temperature range, the activities of SOD and POD in *R. pedestris* were generally higher than in the suitable temperature range; in the lethal high-temperature range, however, SOD and POD activities were generally lower. For example, the SOD activity of Eurygaster austriaca increased significantly at 24–32 °C [43], while the SOD activity of *Ophraella communa* decreased significantly at 44 °C [44], and the POD activity of *Oryzaephilus surinamensis* decreased significantly at 33–45 °C [40]. These trends are consistent with our finding that POD activity in *R. pedestris* decreases in the lethal high-temperature range. Insects upregulate SOD activity to counter oxidative stress caused by elevated temperatures, but as temperatures rise further into the lethal range, extreme heat and oxidative damage may lead to a decline in SOD activity, inhibiting antioxidant capacity [40,44]. This aligns with our observation that SOD activity in *R. pedestris* is generally lower in the lethal high-temperature range than in the suitable temperature range. In the early stage of short-term high-temperature stress, the ROS produced in insects have not yet fully activated the antioxidant system; as stress intensifies, the accumulation of hydrogen peroxide prompts insects to synthesize POD to protect cells from oxidative damage. However, at extreme high temperatures, despite elevated POD activity to counter oxidative damage, insect physiological function is impaired [14,43,44]. Within the sublethal and lethal high-temperature ranges, CAT activity in *R. pedestris* was generally lower than in the suitable temperature range. As short-term high-temperature stress intensifies, the antioxidant system begins to strengthen; although CAT activity does not change significantly, the internal antioxidant system gradually adapts to excess ROS, stabilizing CAT activity and demonstrating the system’s stability [14,41]. However, CAT activity increases under high temperatures in *Chilo suppressalis*, *Toxoptera aurantii*, and *Propylaea japonica* [18,45,46], which contradicts our finding that CAT activity in *R. pedestris* is generally lower in high-temperature ranges. This discrepancy may reflect interspecific differences in antioxidant defense strategies: SOD acts as the primary defense mechanism against ROS damage under high temperatures by catalyzing the conversion of superoxide anions to oxygen and hydrogen peroxide, reducing ROS damage [47]. CAT converts hydrogen peroxide to water and oxygen, effectively preventing its accumulation [48], while POD has broad antioxidant capabilities, participating in the scavenging of hydrogen peroxide and other peroxides [48]. Insects can rapidly adapt to oxidative stress induced by temperature changes by adjusting antioxidant enzyme activities [49]. T-AOC and MDA are important indicators of insect lipid peroxidation and antioxidant capacity, reflecting the balance between oxidative damage and antioxidant defense under high-temperature stress [45,50]. In the sublethal high-temperature range, the T-AOC capacity in *R. pedestris* was disrupted, and the MDA content was generally higher than in the suitable temperature range; in the lethal high-temperature range, the T-AOC capacity was generally lower, while the MDA content remained higher. MDA is a product of lipid peroxidation, reflecting the degree of oxidative damage to cell membranes and other macromolecules [43]. Under short-term high-temperature stress, insects suffer obvious oxidative stress, accompanied by changes in antioxidant-related parameters [36]. This is consistent with our observation that T-AOC decreases and MDA increases in *R. pedestris* under high temperatures. The T-AOC level varies with the activities of antioxidant enzymes (e.g., SOD, CAT, POD), reflecting physiological responses to oxidative stress [36]. However, at extreme high temperatures, insects cannot effectively counter stress, increasing the risk of oxidative damage [43]. In the sublethal and lethal high-temperature ranges, excess ROS accumulation leads to lipid peroxidation of cell membranes, significantly increasing MDA content [36], indicating that oxidative damage in insects intensifies, causing severe damage to cell membranes and other cellular components [8].

The integration of energy substances and antioxidant defense suggests an ‘energy–redox’ network that may underlie the heat stress response of *R. pedestris*. Under sublethal temperatures, ROS accumulation appears to drive preferential energy allocation: carbohydrates accumulate, possibly to store reducing equivalents (NADPH) for the antioxidant system; SOD and POD activities increase, potentially contributing to cellular redox homeostasis; and non-essential functions may be suppressed to conserve adenosine triphosphate (ATP). This protective strategy, however, likely entails a trade-off—reduced CAT activity—and appears to have a thermal limit. Under lethal temperatures (~42–44 °C), if ROS-induced mitochondrial dysfunction occurs, it could theoretically impair ATP synthesis, potentially creating a positive feedback loop. However, this mechanism remains hypothetical pending direct measurements of mitochondrial function and cellular ATP levels. These patterns suggest that both the intensity and duration of heat stress are important determinants of physiological outcomes, highlighting the potential value of incorporating realistic heat wave profiles into predictive models of insect thermal vulnerability [51,52,53,54].

*R. pedestris* is an important agricultural pest [55], and understanding its thermal physiology has practical significance for pest suitability, forecasting, and management. The documented thermal tolerance limits and physiological response patterns suggest that this species possesses a certain capacity to withstand short-term heat events [6,20]. The severe physiological costs at extreme temperatures indicate that heat waves exceeding 40 °C could substantially impact population dynamics, particularly if the exposure duration extends beyond 2–3 h. The observed sexual differences in thermal responses may influence sex ratios and reproductive output under thermal stress, with potential consequences for population growth rates. While the observed physiological changes suggest active stress responses, whether these translate into enhanced survival or fitness under natural heat waves remains to be tested through demographic or field studies. Future research should examine the heritability of thermal tolerance traits and the potential for adaptive evolution in response to selection pressure from increasing heat wave frequency and intensity. While this study provides comprehensive data on short-term high-temperature effects, several limitations should be acknowledged. The laboratory conditions used may not fully capture the complexity of natural thermal environments, including fluctuating temperatures, humidity variations, and interactions with other stressors. Additionally, the focus on adult physiological responses does not address potential critical stage-specific sensitivities or carry-over effects across life stages. Future research should incorporate (1) field-relevant thermal regimes including fluctuating temperatures; (2) multi-stressor approaches combining heat with desiccation or starvation; (3) transcriptomic and metabolomic analyses to elucidate molecular mechanisms underlying the observed physiological changes; and (4) population-level studies to assess thermal tolerance heritability and adaptive potential [56,57,58]. This study was conducted under short-term laboratory conditions and relevant functional validation of the observed physiological responses is lacking; these represent the main limitations of this research. These limitations need to be addressed in further in-depth and long-term field investigations to verify the relevant physiological responses and their ecological relevance in natural environments.

## 5. Conclusions

Short-term high-temperature stress significantly affected energy substances and antioxidant capacity in adult *R. pedestris*. The water loss rate increased with rising temperature and prolonged exposure, reaching a maximum at 44 °C for 4 h. The fat, total sugar, glycogen, and protein contents showed complex temperature- and time-dependent patterns, with sexual differences observed under specific conditions. Antioxidant enzyme activities (SOD, POD, CAT, T-AOC) exhibited significant effects between temperature and exposure time, suggesting activated oxidative stress responses. MDA content generally peaked at moderate temperatures (32–36 °C), which is consistent with temperature-induced lipid peroxidation. These findings suggest that *R. pedestris* adults exhibit certain physiological plasticity in response to sublethal heat stress via changes in metabolic and antioxidant-related parameters, while extreme temperatures (42–44 °C) lead to severe physiological damage. Sex-specific differences in thermal responses are consistent with divergent physiological responses between sexes. This study may provide a basis for future studies exploring the thermal biology and physiological responses to heat stress of this pest species under climate warming scenarios.

## Data Availability

The original contributions presented in this study are included in the article/Supplementary Materials. Further inquiries can be directed to the corresponding authors.

## Supplementary Materials

The following supporting information can be downloaded at: https://www.mdpi.com/article/10.3390/insects17050506/s1. Table S1: The reaction system of determination of total carbohydrate. Table S2: The reaction system of determination of protein. Table S3: The reaction system of determination of superoxide dismutase activity. Table S4: The reaction system of determination of peroxidase activity. Table S5: The reaction system of determination of catalase activity. Table S6: The reaction system of determination of malonic aldehyde activity. Table S7: Statistical analysis of the effects of different temperature and time treatments on water loss rate of *Riptortus pedestris*. Table S8: Statistical analysis of the effects of different temperature and time treatments on fat content of *Riptortus pedestris*. Table S9: Statistical analysis of the effects of different temperature and time treatments on total carbohydrate content of *Riptortus pedestris*. Table S10: Statistical analysis of the effects of different temperature and time treatments on glycogen content of *Riptortus pedestris*. Table S11: Statistical analysis of the effects of different temperature and time treatments on protein content of *Riptortus pedestris*. Table S12: Statistical analysis of the effects of different temperature and time treatments on superoxide dismutase (SOD) activity of *Riptortus pedestris*. Table S13: Statistical analysis of the effects of different temperature and time treatments on peroxidase (POD) activity of *Riptortus pedestris*. Table S14: Statistical analysis of the effects of different temperature and time treatments on catalase (CAT) activity of *Riptortus pedestris*. Table S15: Statistical analysis of the effects of different temperature and time treatments on total antioxidant capacity (T-AOC) of *Riptortus pedestris*. Table S16: Statistical analysis of the effects of different temperature and time treatments on malonic aldehyde (MDA) activity of *Riptortus pedestris*.
