# Supplementary material for: Effects of Temperature and Exposure Duration on Energy Substances and Antioxidant Enzymes in Riptortus pedestris (Hemiptera: Alydidae)
Source: Insects. 2026 May 15;17(5):506. doi: 10.3390/insects17050506 (PMC13206865; doi:10.3390/insects17050506)
Supplement: Supplementary file 1 [file insects-17-00506-s001.zip › insects-4274948-supplementary.pdf]

Supplementary Materials

Effects of Temperature and Exposure Duration on Energy Substances and Antioxidant Enzymes in *Riptortus pedestris* (Hemiptera: Alydidae)

Table S1 The reaction system of determination of total carbohydrate

Samples and reagents	(μL) Volume		
	Sample tube	Standard tube	Blank tube
Sample	30	—	—
Standard	—	30	—
Distille water	—	—	30
Reagent	30	30	30
Distille water	180	180	180

Table S2 The reaction system of determination of protein

Samples and reagents	(μL) Volume		
	Sample tube	Standard tube	Blank tube
Sample	10	—	—
Standard	—	10	—
Distille water	—	—	10
Reagent	250	250	250

Table S3 The reaction system of determination of superoxide dismutase activity

Samples and reagents	(μL) Volume		
	Sample tube	Blank1 tube	Blank2 tube
Sample	20	—	—
SOD detection buffer	—	20	40
Reagent 1	160	160	160
Reagent 2	20	20	—

Table S4 The reaction system of determination of peroxidase activity

Samples and reagents	(μL) Volume		
	Sample tube	Standard tube	Blank tube
Sample	50	—	—
Standard	—	50	—
Distilled water	—	—	50
Reagent 1	50	50	50
Reagent 2	50	50	50
Reagent 3	50	50	50

Table S5 The reaction system of determination of catalase activity

Samples and reagents	(μL) Volume	
	Sample tube	Control tube
Sample	50	—
Reagent 1	100*	100*
Reagent 2	100	100
Sample	—	50

* After adding the reagent 1, incubate at 37°C for 1 minute

Table S6 The reaction system of determination of malonic aldehyde activity

Samples and reagents	(μL) Volume		
	Sample tube	Standard tube	Blank tube
Sample	100	—	—
Standard	—	100	—
Distille water	—	—	100
Reagent	200	200	200

Table S7 Statistical analysis of the effects of different temperature and time treatments on water loss rate of *Riptortus pedestris*.

Factor	♀			♂		
	F	*p*	*Partial* η^2	*F*	*p*	*Partial* $\eta 2$
Temperature	110.43	<0.001	0.67	66.37	<0.001	0.56
Exposure duration	29.95	<0.001	0.25	14.31	<0.001	0.14
Temperature×Exposure duration	10.56	<0.001	0.37	4.84	<0.001	0.22

Table S8 Statistical analysis of the effects of different temperature and time treat ments on fat content of *Riptortus pedestris*.

Factor	♀			♂		
	F	*p*	*Partial η2*	*F*	*p*	*Partial η 2*
Temperature	30.08	<0.001	0.76	25.10	<0.001	0.72
Exposure duration	4.39	0.008	0.22	12.93	<0.001	0.45
Temperature×Exposure duration	10.25	<0.001	0.76	14.65	<0.001	0.82

Table S9 Statistical analysis of the effects of different temperature and time treat ments on total carbohydrate content of *Riptortus pedestris*.

Factor	♀			♂		
	F	*p*	*Partial η2*	*F*	*p*	*Partial η2*
Temperature	494.97	<0.001	0.95	1268.89	<0.001	0.98
Exposure duration	485.86	<0.001	0.92	280.13	<0.001	0.88
Temperature×Exposure duration	207.83	<0.001	0.96	280.48	<0.001	0.97

Table S10 Statistical analysis of the effects of different temperature and time treatments on glycogen content of *Riptortus pedestris*.

Factor	♀			♂		
	F	*p*	*Partial η2*	*F*	*p*	*Partial η2*
Temperature	64.57	<0.001	0.76	407.89	<0.001	0.95
Exposure duration	6.39	<0.001	0.15	254.67	<0.001	0.89
Temperature×Exposure duration	4.02	<0.001	0.37	132.72	<0.001	0.95

Table S11 Statistical analysis of the effects of different temperature and time treatments on protein content of *Riptortus pedestris*.

Factor	♀			♂		
	F	*p*	*Partial η2*	*F*	*p*	*Partial η2*
Temperature	148.49	<0.001	0.93	95.95	<0.001	0.91
Exposure duration	62.21	<0.001	0.81	253.52	<0.001	0.95
Temperature×Exposure duration	76.89	<0.001	0.95	106.16	<0.001	0.97

Table S12 Statistical analysis of the effects of different temperature and time treatments on superoxide dismutase (SOD) activity of *Riptortus pedestris*.

Factor	♀			♂		
	F	*p*	*Partial η2*	*F*	*p*	*Partial η2*
Temperature	144.04	<0.001	0.93	63.31	<0.001	0.82
Exposure duration	154.56	<0.001	0.91	38.24	<0.001	0.67
Temperature×Exposure duration	79.87	<0.001	0.95	15.45	<0.001	0.77

Table S13 Statistical analysis of the effects of different temperature and time treatments on peroxidase (POD) activity of *Riptortus pedestris*.

Factor	♀			♂		
	F	*p*	*Partial η2*	*F*	*p*	*Partial η2*
Temperature	94.56	<0.001	0.81	46.36	<0.001	0.68
Exposure duration	0.62	0.61	0.02	77.19	<0.001	0.72
Temperature×Exposure duration	5.49	<0.001	0.42	16.15	<0.001	0.69

Table S14 Statistical analysis of the effects of different temperature and time treatments on catalase (CAT) activity of *Riptortus pedestris*.

Factor	♀			♂		
	F	*p*	*Partial η2*	*F*	*p*	*Partial η2*
Temperature	767.74	<0.001	0.99	114.79	<0.001	0.93
Exposure duration	91.09	0.61	0.86	23.32	<0.001	0.60
Temperature×Exposure duration	105.48	<0.001	0.97	26.67	<0.001	0.90

Table S15 Statistical analysis of the effects of different temperature and time treatments on total antioxidant capacity (T-AOC) of *Riptortus pedestris*.

Factor	♀			♂		
	F	*p*	*Partial η2*	*F*	*p*	*Partial η2*
Temperature	79.67	<0.001	0.85	16.49	<0.001	0.53
Exposure duration	36.51	0.61	0.60	42.58	<0.001	0.64
Temperature×Exposure duration	10.53	<0.001	0.67	50.76	<0.001	0.91

Table S16 Statistical analysis of the effects of different temperature and time treatments on malonic aldehyde (MDA) activity of *Riptortus pedestris*.

Factor	♀			♂		
	F	*p*	*Partial η2*	*F*	*p*	*Partial η2*
Temperature	42.53	<0.001	0.76	15.96	<0.001	0.54
Exposure duration	15.82	0.61	0.42	1.86	<0.001	0.08
Temperature×Exposure duration	4.19	<0.001	0.49	0.33	<0.001	0.22
